# The Mexican Spanish version of the Juvenile Arthritis Multidimensional Assessment Report (JAMAR)

**DOI:** 10.1007/s00296-018-3961-0

**Published:** 2018-04-07

**Authors:** Raul Gutierrez-Suarez, Ruben Burgos-Vargas, Gabriela Burgos-Martinez, Alessandro Consolaro, Francesca Bovis, Nicolino Ruperto, For the Paediatric Rheumatology International Trials Organisation (PRINTO)

**Affiliations:** 1 Departamento de Reumatología, Hospital General de Mexico, Dr Balmis 148, 06726 Mexico City, Mexico; 2 Rockyview General Hospital, Calgary, Canada; 3 Clinica Pediatrica e Reumatologia, Paediatric Rheumatology International Trials Organisation (PRINTO), Istituto Giannina Gaslini, Via Gaslini 5, 16147 Genoa, Italy; 4 Dipartimento di Pediatria, Università di Genova, Genoa, Italy

**Keywords:** Juvenile idiopathic arthritis, Disease status, Functional ability, Health related quality of life, JAMAR

## Abstract

The Juvenile Arthritis Multidimensional Assessment Report (JAMAR) is a new parent/patient reported outcome measure that enables a thorough assessment of the disease status in children with juvenile idiopathic arthritis (JIA). We report the results of the cross-cultural adaptation and validation of the parent and patient versions of the JAMAR in the Mexican Spanish language. The reading comprehension of the questionnaire was tested in 10 JIA parents and patients. Each participating centre was asked to collect demographic, clinical data and the JAMAR in 100 consecutive JIA patients or all consecutive patients seen in a 6-month period and to administer the JAMAR to 100 healthy children and their parents. The statistical validation phase explored descriptive statistics and the psychometric issues of the JAMAR: the 3 Likert assumptions, floor/ceiling effects, internal consistency, Cronbach’s alpha, interscale correlations, test–retest reliability, and construct validity (convergent and discriminant validity). A total of 100 JIA patients (16% systemic, 16% oligoarticular, 30% RF positive polyarthritis, 38% other categories) and 99 healthy children, were enrolled at the paediatric rheumatology department of the Hospital General de Mexico. The JAMAR components discriminated well healthy subjects from JIA patients. However, there was no significant difference between healthy subjects and their affected peers in school related problem variable. All JAMAR components revealed good psychometric performances. In conclusion, the Mexican Spanish version of the JAMAR is a valid tool for the assessment of children with JIA and is suitable for use both in routine clinical practice and clinical research.

## Introduction

The aim of the present study was to cross-culturally adapt and validate the Mexican Spanish parent, child/adult version of the Juvenile Arthritis Multidimensional Assessment Report (JAMAR) [1] in patients with juvenile idiopathic arthritis (JIA). The JAMAR assesses the most relevant parent/patient reported outcomes in JIA, including overall well-being, functional status, health related quality of life (HRQoL), pain, morning stiffness, disease activity/status/course, articular and extra-articular involvement, drugs side effects/compliance and satisfaction with illness outcome.

This project was part of a larger multinational study conducted by the Paediatric Rheumatology International Trials Organisation (PRINTO) [2] aimed to evaluate the Epidemiology, Outcome and Treatment of Childhood Arthritis (EPOCA) in different geographic areas [3].

We report herein the results of the cross-cultural adaptation and validation of the parent and patient versions of the JAMAR in the Mexican Spanish language.

## Materials and methods

The methodology employed has been described in detail in the introductory paper of the supplement [4]. In brief, it was a cross-sectional study of JIA children, classified according to the ILAR criteria [5, 6] and enrolled from October 2011 to June 2012. Children were recruited after Ethics Committee approval and consent from at least one parent.

### The JAMAR

The JAMAR [1] includes the following 15 sections:

1. Assessment of physical function (PF) using 15-items in which the ability of the child to perform each task is scored as follows: 0 = without difficulty, 1 = with some difficulty, 2 = with much difficulty, 3 = unable to do and not applicable if it was not possible to answer the question or the patient was unable to perform the task due to their young age or to reasons other than JIA. The total PF score ranges from 0 to 45 and has 3 components: PF-lower limbs (PF-LL); PF-hand and wrist (PF-HW) and PF-upper segment (PF-US) each scoring from 0 to 15 [7]. Higher scores indicating higher degree of disability [8–10];
2. Rating of the intensity of the patient’s pain on a 21-numbered circle visual analogue scale (VAS) [11];
3. Assessment of the presence of joint pain or swelling (present/absent for each joint);
4. Assessment of morning stiffness (present/absent);
5. Assessment of extra-articular symptoms (fever and rash) (present/absent);
6. Rating of the level of disease activity on a 21-circle VAS;
7. Rating of disease status at the time of the visit (categorical scale);
8. Rating of disease course from previous visit (categorical scale);
9. Checklist of the medications the patient is taking (list of choices);
10. Checklist of side effects of medications;
11. Report of difficulties with medication administration (list of items);
12. Report of school/university/work problems caused by the disease (list of items);
13. Assessment of HRQoL, through the Physical Health (PhH), and Psychosocial Health (PsH) subscales (5 items each) and a total score. The four-point Likert response, referring to the prior month, are ‘never’ (score = 0), ‘sometimes’ (score = 1), ‘most of the time’ (score = 2) and ‘all the time’ (score = 3). A ‘not assessable’ column was included in the parent version of the questionnaire to designate questions that cannot be answered because of developmental immaturity. The total HRQoL score ranges from 0 to 30, with higher scores indicating worse HRQoL. A separate score for PhH and PsH (range 0–15) can be calculated [12–14];
14. Rating of the patient’s overall well-being on a 21-numbered circle VAS;
15. A question about satisfaction with the outcome of the illness (Yes/No) [15].

The JAMAR is available in three versions, one for parent proxy-report (child’s age 2–18), one for child self-report, with the suggested age range of 7–18 years, and one for adults.

### Cross cultural adaptation and validation

The process of cross-cultural adaptation was conducted according to international guidelines with 2–3 forward and backward translations. In those countries for which the translation of JAMAR had been already cross-cultural adapted in a similar language (i.e. Spanish in South American countries), only the probe technique was performed. Reading comprehension and understanding of the translated questionnaires were tested in a probe sample of 10 JIA parents and 10 patients.

Each participating centre was asked to collect demographic, clinical data and the JAMAR in 100 consecutive JIA patients or all consecutive patients seen in a 6-month period and to administer the JAMAR to 100 healthy children and their parents.

The statistical validation phase explored the descriptive statistics and the psychometric issues [16]. In particular, we evaluated the following validity components: the first Likert assumption [mean and standard deviation (SD) equivalence]; the second Likert assumption or equal items-scale correlations (Pearson *r*: all items within a scale should contribute equally to the total score); third Likert assumption (item internal consistency or linearity for which each item of a scale should be linearly related to the total score that is 90% of the items should have Pearson *r* ≥ 0.4); floor/ceiling effects (frequency of items at lower and higher extremes of the scales, respectively); internal consistency, measured by the Cronbach’s alpha, interscale correlation (the correlation between two scales should be lower than their reliability coefficients, as measured by Cronbach’s alpha); test–retest reliability or intra-class correlation coefficient (reproducibility of the JAMAR repeated after 1 or 2 weeks); and construct validity in its two components: the convergent or external validity which examines the correlation of the JAMAR sub-scales with the 6 JIA core set variables, with the addition of the parent assessment of disease activity and pain by the Spearman’s correlation coefficients (*r*) [17] and the discriminant validity, which assesses whether the JAMAR discriminates between the different JIA categories and healthy children [18].

Quantitative data were reported as medians with 1st and 3rd quartiles and categorical data as absolute frequencies and percentages.

The complete Mexican Spanish parent and patient versions of the JAMAR are available upon request to PRINTO.

## Results

### Cross cultural adaptation

The Mexican Spanish JAMAR was fully cross-culturally adapted from the standard English version with 3 forward and 2 backward translations with a concordance for 118/123 translations lines (95.9%) for the parent version and 120/120 lines (100%) for the child version.

All 123 lines of the parent version of the JAMAR were understood by at least 80% of the 10 parents tested (median = 100%; range 80–100%). All the 120 lines of the patient version of the JAMAR were understood by at least 80% of the children (median = 100%; range 80–100%). The texts of the parent JAMAR and of the child JAMAR were unmodified after the probe technique.

### Demographic and clinical characteristics of the subjects

A total of 100 JIA patients and 99 healthy children (total of 199 subjects), were enrolled. Patients recruited for the study were mainly attended the Hospital General de México, but there also referrals from the Shriners Hospital in Mexico City and the Clinic for the Diagnosis and Treatment of the Rheumatic Diseases (CLIDITER = Clinica para el Diagnostico y Tratamiento de las Enfermedades Reumaticas). In the 100 JIA subjects, the JIA categories were 16% with systemic arthritis, 16% with oligoarthritis, 20% with RF negative polyarthritis, 30% with RF positive polyarthritis, 1% with psoriatic arthritis, 16% with enthesitis related arthritis and 1% with undifferentiated arthritis (Table 1).

**Table 1:** Descriptive statistics (medians, 1st–3rd quartiles or absolute frequencies and %) for the 100 JIA patients

	Systemic (*N* = 16)	Oligoarthritis (*N* = 16)	RF− Poly-arthritis (*N* = 20)	RF+ Poly-arthritis (*N* = 30)	Psoriatic arthritis (*N* = 1)	Enthesitis related arthritis (*N* = 16)	Undifferentiated arthritis (*N* = 1)	All JIA patients (*N* = 100)	Healthy (*N* = 99)
Female	6 (37.5%)	12 (75%)	14 (70%)	27 (90%)	1 (100%)	1 (6.3%)	0 (0%)	61 (61%)	53 (53.5%)
Age at visit	14.1 (9.2–15.5)	8.9 (7.8–13.5)	13.9 (11.2–15.1)	13.6 (11.6–15.3)	10.5 (10.5–10.5)	15 (14.3–16.2)	8.4 (8.4–8.4)	13.8 (10.5–15.2)*	11.5 (9.9–13.2)*
Age at onset	5.3 (3.5–7.3)	5.1 (2.8–6.7)	8.5 (6.8–10.3)	8.3 (6.3–11.1)	6 (6–6)	10.8 (6.9–12.9)	5.4 (5.4–5.4)	7.1 (5.5–10.7)**	
Disease duration	7.1 (5.2–9)	4.6 (1.6–6.5)	4.1 (2.9–6.6)	3.1 (1.8–6.7)	4.5 (4.5–4.5)	3.6 (1.5–6.9)	3 (3–3)	4.4 (2.7–6.9)*	
ESR	22 (15–28)	10 (10–13.5)	11 (5–18)	28 (15–32)	20 (20–20)	20.5 (12.5–28)	12 (12–12)	16 (10–28)*	
MD VAS	1 (0–2)	1 (0–2)	1 (0–2)	2.5 (2–5)	3 (3–3)	0.5 (0–3)	–	1.5 (0–3)	
No. swollen joints	0 (0–3)	0.5 (0–1)	0 (0–1)	4 (1–10)	0 (0–0)	0 (0–1)	1 (1–1)	0.5 (0–3.5)*	
No. joints with pain	1.5 (0–4.5)	0 (0–1)	0 (0–3.5)	4.5 (2–11)	1 (1–1)	1 (0–1)	1 (1–1)	1 (0–5)*	
No. joints with LOM	5.5 (4–15.5)	1 (1–3.5)	7 (5–14.5)	20.5 (9–31)	1 (1–1)	1.5 (0–2.5)	5 (5–5)	5.5 (1–17.5)^#^	
No. active joints	1.5 (0–5)	0.5 (0–1)	0 (0–2.5)	4 (1–10)	1 (1–1)	0.5 (0–1)	1 (1–1)	1 (0–4.5)*	
Active systemic features	0 (0%)	0 (0%)	0 (0%)	0 (0%)	0 (0%)	0 (0%)	0 (0%)	0 (0%)	
ANA status	1 (6.3%)	6 (37.5%)	8 (40%)	0 (0%)	1 (100%)	0 (0%)	0 (0%)	16 (16%)*	
Uveitis	0 (0%)	2 (12.5%)	0 (0%)	0 (0%)	0 (0%)	1 (6.3%)	0 (0%)	3/99 (3%)	
PF Total Score	8 (4–21)	1.5 (0–4.5)	5.5 (1.5–12)	12 (8–21)	6 (6–6)	4 (1–8)	10 (10–10)	7 (2–14)^#^	0 (0–0)^#^
Pain VAS	1.8 (0.3–3.3)	0.3 (0–1.5)	0 (0–2.5)	2.8 (1–5)	5.5 (5.5–5.5)	1.8 (0.8–5.8)	5 (5–5)	2 (0–4)*	0 (0–0)^#^
Disease Activity VAS	1.5 (0–3.3)	0 (0–1.5)	0.3 (0–2.5)	3 (1.5–5)	3 (3–3)	2 (0.3–5.8)	3 (3–3)	1.5 (0–3.8)*	
Well-being VAS	2.3 (0.3–3.5)	0 (0–1.3)	1 (0–3.5)	2.8 (1.5–5)	4.5 (4.5–4.5)	2.5 (1–5)	3 (3–3)	2 (0–4.3)	
HRQoL PhH	4.5 (2–7.5)	0.5 (0–4)	3 (0.5–5.5)	6 (4–8)	6 (6–6)	3 (1–6)	9 (9–9)	4 (1–6)*	0 (0–0)^#^
HRQoL PsH	3 (1–5.5)	1 (0–5.5)	2.5 (1.5–5)	4 (2–7)	7 (7–7)	3 (0–4.5)	5 (5–5)	3 (1–5.5)	1 (0–2)^#^
HRQoL total score	7.5 (5–15)	2 (1–8)	5.5 (4–8.5)	9.5 (6–16)	13 (13–13)	7 (2–10)	14 (14–14)	7 (4–12)*	1 (0–2)^#^
Pain/swell. in > 1 joint	10 (62.5%)	6 (37.5%)	8 (40%)	24 (80%)	1 (100%)	12 (75%)	1 (100%)	62 (62%)*	0 (0%)^#^
Morning stiffness > 15 min	9 (56.3%)	4 (25%)	5 (25%)	17 (56.7%)	1 (100%)	8 (50%)	1 (100%)	45 (45%)	1 (1.1%)^#^
Subjective remission	8 (50%)	6 (37.5%)	9 (45%)	24 (80%)	1 (100%)	11 (68.8%)	1 (100%)	60 (60%)*	
In treatment	13 (81.3%)	11 (68.8%)	16 (80%)	30 (100%)	1 (100%)	16 (100%)	1 (100%)	88 (88%)*	
Reporting side effects	4/13 (30.8%)	3/11 (27.3%)	3/16(18.8%)	5 (16.7%)	1 (100%)	6 (37.5%)	0 (0%)	22/88 (25%)	
Taking medication regularly	10/13 (76.9%)	11/11 (100%)	14/16 (87.5%)	24 (80%)	1 (100%)	14 (87.5%)	0 (0%)	74/88 (84.1%)	
With problems attending school	1/11 (9.1%)	0 (0%)	2/16 (12.5%)	3/23 (13%)	–	0 (0%)	–	6/71 (8.5%)	2 (2.4%)
Satisfied with disease outcome	4 (25%)	9 (56.3%)	11 (55%)	6 (20%)	0 (0%)	2 (12.5%)	0 (0%)	32 (32%)*	

Data related to the JAMAR refers to the 100 JIA patients and to the 93 healthy subjects for whom the questionnaire has been completed by the parents

*JAMAR* Juvenile Arthritis Multidimensional Assessment Report, *ESR* erythrocyte sedimentation rate, *MD* medical doctor, *VAS* visual analogue scale (score 0–10; 0 = no activity, 10 = maximum activity), *LOM* limitation of motion, *ANA* anti-nuclear antibodies, *PF* physical function (total score ranges from 0 to 45), *HRQoL* health related quality of life (total score ranges from 0 to 30), *PhH* physical health (total score ranges from 0 to 15), *PsH* psychosocial health (total score ranges from 0 to 15)

*p* values refers to the comparison of the different JIA categories or to JIA versus healthy. *p < 0.05 **p < 0.001 ^**#**^*p* < 0.0001

A total of 193/199 (97%) subjects had the parent version of the JAMAR completed by a parent (100 from parents of JIA patients and 93 from parents of healthy children). The JAMAR was completed by 181/193 (93.8%) mothers and 12/193 (6.2%) fathers. The child version of the JAMAR was completed by all the 199 children age 5 or older. Also patients younger than 7 years old, capable to assess their personal condition and able to read and write, were asked to fill in the patient version of the questionnaire.

### Discriminant validity

The JAMAR results are presented in Table 1, including the scores [median (1st−3rd quartile)] obtained for the PF, the PhH, the PsH subscales and total score of the HRQoL scales. The JAMAR components discriminated well between healthy subjects and JIA patients.

In summary, the JAMAR revealed that JIA patients had a greater level of disability and pain, as well as a lower HRQoL than their healthy peers. However, there was no significant difference between healthy subjects and their affected peers in school related problem variable.

### Psychometric issues

The main psychometric properties of both parent and child versions of the JAMAR are reported in Table 2. The following results section refers mainly to the parent’s version findings, unless otherwise specified.

**Table 2:** Main psychometric characteristics between the parent and child version of the JAMAR

	Parent (*N* = 100/193)	Child (*N* = 100/199)
Missing values (1st–3rd quartiles)	No missing values	No missing values
Response pattern	PF and HRQoL positively skewed	PF and HRQoL positively skewed
Floor effect, median		
PF	56.0%	69.0%
HRQoL PhH	38.0%	45.0%
HRQoL PsH	47.0%	54.0%
Pain VAS	28.0%	38.0%
Disease activity VAS	30.0%	38.0%
Well-being VAS	27.0%	34.0%
Ceiling effect, median		
PF	3.0%	2.0%
HRQoL PhH	9.0%	6.0%
HRQoL PsH	4.0%	2.0%
Pain VAS	1.0%	1.0%
Disease activity VAS	1.0%	0.0%
Well-being VAS	1.0%	1.0%
Items with equivalent item-scale correlation	93% for PF, 100% for HRQoL	93% for PF, 100% for HRQoL
Items with items-scale correlation ≥ 0.4	100% for PF, 100% for HRQoL	93% for PF, 100% for HRQoL
Cronbach’s alpha		
PF-LL	0.95	0.92
PF-HW	0.97	0.95
PF-US	0.91	0.79
HRQoL-PhH	0.92	0.91
HRQoL-PsH	0.85	0.83
Items with item-scale correlation lower than the Cronbach alpha	100% for PF, 100% for HRQoL	100% for PF, 100% for HRQoL
Test–retest intraclass correlation		
PF total score	0.88	0.82
HRQoL-PhH	0.91	0.66
HRQoL-PsH	0.80	0.88
Spearman correlation with JIA core-set variables, median		
PF	0.6	0.6
HRQoL PhH	0.7	0.7
HRQoL PsH	0.4	0.4
Pain VAS	0.5	0.6
Disease activity VAS	0.5	0.5
Well-being VAS	0.6	0.5

*JAMAR* Juvenile Arthritis Multidimensional Assessment Report, *JIA* juvenile idiopathic arthritis, *VAS* visual analogue scale, *PF* physical function, *HRQoL* health related quality of life, *PhH* physical health, *PsH* psychosocial health, *PF-LL* PF-lower limbs, *PF-HW* PF-hand and wrist, *PF-US* PF-upper segment

### Descriptive statistics (first Likert assumption)

There were no missing results for all JAMAR items, since data were collected through a web-based system that did not allow to skip answers and input of null values. The response pattern for both PF and HRQoL was positively skewed toward normal functional ability and normal HRQoL. All response choices were used for the different HRQoL items, whereas a reduced number of response choices was used for PF item 15.

The mean and SD of the items within a scale were roughly equivalent for the PF and for the HRQoL items, except for PF item 15 (data not shown). The median number of items marked as not applicable was 0% (0–0%) for the PF and 0.5% (0–1%) for the HRQoL.

### Floor and ceiling effect

The median floor effect was 56% (47–74%) for the PF items, 38% (32–40%) for the HRQoL PhH items, and 47% (44–48%) for the HRQoL PsH items. The median ceiling effect was 3% (2–8%) for the PF items, 9% (8–13%) for the HRQoL PhH items, and 4% (1–6%) for the HRQoL PsH items. The median floor effect was 28% for the pain VAS, 30% for the disease activity VAS and 27% for the well-being VAS. The median ceiling effect was 1% for the pain VAS, 1% for the disease activity VAS and 1% for the well-being VAS.

### Equal items-scale correlations (second Likert assumption)

Pearson items-scale correlations corrected for overlap were roughly equivalent for items within a scale for 93% of the PF items, with the exception of PF item 15, and for 100% of the HRQoL items.

### Items internal consistency (third Likert assumption)

Pearson items-scale correlations were ≥ 0.4 for 100% of items of the PF and 100% of items of the HRQoL.

### Cronbach’s alpha internal consistency

Cronbach’s alpha was 0.95 for PF-LL, 0.97 for PF-HW, 0.91 for PF-US. Cronbach’s alpha was 0.92 for HRQoL-PhH and 0.85 for HRQoL-PsH.

### Interscale correlation

The Pearson correlation of each item of the PF and the HRQoL with all items included in the remaining scales of the questionnaires was lower than the Cronbach’s alpha.

### Test–retest reliability

Reliability was assessed in 10 JIA patients, by re-administering both versions (parent and child) of the JAMAR after a median of 6.5 days (6–7 days). The intraclass correlation coefficients (ICC) for the PF total score showed an almost perfect reproducibility (ICC = 0.88). The ICC for the HRQoL PhH and for the HRQoL PsH showed an almost perfect reproducibility (ICC = 0.91 and ICC = 0.80, respectively).

### Convergent validity

The Spearman correlation of the PF total score with the JIA core set of outcome variables ranged from 0.6 to 0.7 (median = 0.6). The PF total score best correlation was observed with the number of joints with the limitation of motion (*r* = 0.7, *p* < 0.001). For the HRQoL, the median correlation of the PhH with the JIA core set of outcome variables ranged from 0.6 to 0.7 (median = 0.7), whereas for the PsH ranged from 0.3 to 0.5 (median = 0.4). The PhH showed the best correlation with the parent’s assessment of pain (*r* = 0.8, *p* < 0.001) and the PsH with the parent global assessment of well-being (*r* = 0.6, *p* < 0.001). The median correlations between the pain VAS, the well-being VAS, and the disease activity VAS and the physician-centred and laboratory measures were 0.5 (0.4–0.6), 0.5 (0.4–0.6), 0.6 (0.5–0.6), respectively.

## Discussion

In this study, the Mexican Spanish version of the JAMAR was cross-culturally adapted from the original standard English version with three forward and two backward translations. According to the results of the validation analysis, the Mexican Spanish parent and patient versions of the JAMAR possess satisfactory psychometric properties. The disease-specific components of the questionnaire discriminated well between patients with JIA and healthy controls. Notably, there was no significant difference between the healthy subjects and their affected peers in the school related problem variable. This finding indicates that children with JIA adapt well to the consequences of JIA. The PF total score proved to discriminate between the different JIA subtypes with children with RF positive poly-arthritis having a higher degree of disability.

Psychometric performances were good for all domains of the JAMAR and the overall internal consistency was excellent for all the domains.

In the external validity evaluation, the Spearman’s correlations of the PF and HRQoL scores with JIA core set parameters ranged from moderate to strong.

The results obtained for the parent version of the JAMAR are very similar to those obtained for the child version, which suggests that children are equally reliable proxy reporters of their disease and health status as their parents. The JAMAR is aimed to evaluate the side effects of medications and school attendance, which are other dimensions of daily life that were not previously considered by other HRQoL tools. This may provide useful information for intervention and follow-up in health care.

In conclusion, the Mexican Spanish version of the JAMAR was found to have satisfactory psychometric properties and it is, thus, a reliable and valid tool for the multidimensional assessment of children with JIA.
